# Early sexual debut and risky sex in young adults: the role of low self-control

**DOI:** 10.1186/s12889-019-7734-9

**Published:** 2019-11-08

**Authors:** B. M. Magnusson, A. Crandall, K. Evans

**Affiliations:** Department of Public Health, Brigham Young University, 4103 LSB, Provo, UT 84602 USA

**Keywords:** Sexual behavior, Self-control, Executive function, Sexual debut, Sexual initiation

## Abstract

**Background:**

The purpose of this study was to examine the role of low self-control as a mediator or moderator between early age at sexual debut and risky sexual behavior in young adulthood.

**Methods:**

Data on 5734 male and female *Add* Health participants were used. Self-control (waves 1 & 3), age at sexual debut (wave 3) and risky sexual behavior (wave 4) were used in a structural equation modeling framework to assess the relationships of interest.

**Results:**

Approximately 17% of respondents were < 15 years at first sexual intercourse. Among females only, both early age at first intercourse (Parent-report: *z* = 5.08, *p* < .001; Self-report: *z* = 2.05, *p* < .05) and low self-control at wave 3 (Parent-report: *z* = 2.30, *p* < .05; Self-report: *z* = 2.31, *p* < .05) mediated the relationship between low self-control at wave 1 and risky sexual behaviors in young adulthood. Similarly in the male-only model, both early age at first intercourse (Parent-report: *z* = 2.92, *p* < .01; Self-report: *z* = 3.04, *p* < .01) and low self-control at wave 3 (Parent-report: *z* = 1.99, *p* < .05; Self-report: *z* = 3.15, *p* < .01) mediated the relationship between low self-control and risky sexual behaviors in young adulthood. There was evidence of moderation in the male-only model (− 0.26, *p* < .01), such that lower impulsivity strengthened the relationship between early sex and risky sex.

**Conclusions:**

This study confirms the role of executive functions in sexual behaviors and suggests that interventions aimed at improving self-control may be beneficial in reducing risky sexual behavior.

## Background

The onset of sexual activity during adolescence is a normative developmental milestone. By age 19, nearly 70% of both males and females report ever having had sexual intercourse [1]. However, sexual debut occurring at an earlier than normative age (typically < 15 years) is associated with engagement in risky sexual behaviors in adolescence and throughout adulthood. Early sexual experience is associated with increased rates of sexually transmitted infections [2], more sexual partners [3], inconsistent use of contraceptives [4], unintended pregnancy [5], concurrency, and sex with risky partners [6] in adulthood. An estimated 16% of US adolescents have had vaginal intercourse by their 15th birthday [7]. The probability of early sexual debut differs by sex and race/ethnicity. For all races, males have a higher probability of early debut than females. African American and Hispanic youths have a higher probability of early debut than their White and Asian counterparts [8]. While decades of research have linked early debut with risky behaviors, the mechanism of action is not fully understood.

It is suggested that low self-control (e.g. impulsivity or low inhibitory control) may explain the association between timing of sexual debut and risky sexual behavior. Low self-control, specifically impulsivity, has been associated with early sexual initiation [9] and with risky sexual behaviors, including sex while intoxicated, infrequent use of condoms, engaging in sex with strangers, having multiple partners and a history of sexually transmitted disease (STD) [10, 11]. It is suggested that low self-control in adolescence, at least partially, explains selection into early sex [9]. It is further hypothesized that low self-control, present in adolescence, may persist throughout adulthood continuing to influence involvement in risky sexual behaviors and possibly compounding the effects of early sex on later risky sex.

Gender differences in the development of executive functions and the timing and social acceptance of sexual activity exist. For example, girls on average develop more advanced executive functioning skills 1–2 years earlier than males during adolescence [12]. In general males have lower levels of impulse control and higher levels of sensation seeking [13]. Additionally, the level of impulsive behavior [14] and the influence of impulsivity on specific health behaviors may vary by gender [15]. Sexual scripting theory is the application of social scripts to sexual behavior [16]. Sexual scripts, specify the acceptability of sexual behavior in specific times, places, and circumstance and provide individuals acting within those scripts with guidelines for acceptable expression of sexuality. Within Western cultures, social scripts related to sexuality differ markedly by gender, a so-called “sexual double-standard” [17]. These scripts are generally more accepting of sexual expression for males than for females. Consistent with sexual scripting, across all races and ethnicities, US males have a higher probability of early sexual initiation [8]. Social expectations promote sexual expression and behavior for males and restrict sexual expression and behaviors for females. Specifically, a 2016 study examining peer acceptance surrounding sexual initiation observed that females pay a social penalty in terms of peer acceptance over time for engaging in sex, while males receive a social reward in peer acceptance for engaging in sex [18]. Research has also shown that among emerging adults, women perceive that their gender affects their thoughts and feelings about sex [19]. These gendered norms, suggest that it is important to evaluate whether any role of impulsivity in the associations between early sexual debut and risky sexual behavior in adulthood differs by gender.

### Theoretical model and aims

Social Cognitive Theory (SCT) serves as the theoretical basis for this study [20]. SCT proposes that a person’s capacity to self-regulate influences has a significant effect on their behavior. Based on this theory, we would expect that impulsivity/low self-control would be strongly predictive of a younger age at first sex and later engagement in risky sexual behaviors. Further, we would expect that the association between early sex and later risky sexual behaviors would be weaker among those who develop better impulse control (less impulsivity) than among those who are more impulsive. The purposes of this study are 1) to examine the effect of low self-control and impulsivity on early sexual debut and risky sexual behavior in adulthood and 2) to determine if these relationships vary by gender.

We hypothesized that low self-control in wave 1 would be associated with early age at sexual debut in wave 3 and that low self-control in wave 3 would be associated with risky sexual behavior in wave 4. We expected that we would observe persistence of low self-control over time such that low self-control in wave 1 would be predictive of impulsivity at wave 3. Additionally, we hypothesized that early age at sexual debut would mediate the relationship between low self-control at wave 1 and risky sexual behavior in wave 4. We further hypothesized, that low self-control in wave 3 would moderate the relationship between early sex and risky sexual behavior such that the relationship between early age at sexual debut and risky sexual behaviors would be stronger among those with lower self-control. Finally, we hypothesized that these relationships may vary by gender.

The purpose of this study is to examine self-control as a mechanism of action that may partially explain the observed associations between early sexual initiation and sexual risk taking in adulthood. The plurality of the extant research on the relationship between early sexual experience and risky sexual behaviors has examined sexual risk taking that is more proximal to sexual initiation (e.g. condomless sex in adolescence). Although research does identify associations between early sexual experience and sexual risk taking in adulthood, most of these studies have relied on cross-sectional data. Few longitudinal datasets exist which span the entire period from early adolescence into adulthood. The National Longitudinal Study of Adolescent to Adult (*Add*) Health, used for this study, allows for measurement of self-control preceding sexual initiation and before measuring adult sexual experience. Additionally, we used structural equation modeling to analyze the data, allowing us to examine multiple pathways simultaneously.

## Methods

Data for 3032 female and 2702 male participants from three waves of The National Longitudinal Study of Adolescent to Adult (*Add*) Health were used for this study [21]. *Add* Health is an ongoing study that began in the mid-1990s with a nationally representative sample of students in grades 7–12. Four waves of data collection were completed between 1995 and 2007–2008. *Add* Health collects a wide range of data to assess the relationships between behavior and environment with health. We included data from waves 1, 3, and 4 in our analysis. Respondent’s for whom age at first sex was missing at wave 3 were excluded from the analysis.

### Measures

The analytic measures included self-control at two time points, early sexual initiation and risky sexual behavior in adulthood as delineated in the subsequent paragraphs. All constructs were derived from data in waves 1, 3 and 4 of *Add* Health.

#### Self-control

Measures of self-control were modeled after the approach of Beaver, Ratchford & Ferguson [22]. Two measures of low self-control in wave one were used 1) parent report of adolescent low self-control and 2) adolescent self-report of low self-control. Parent report of adolescent low self-control was comprised of four questions (e.g., “Does your child have a bad temper?”). Adolescent self-report of low self-control included four questions inquiring about difficulty paying attention, completing homework and getting along with teachers and peers (e.g., “Do you have trouble paying attention in school?”). For both the parent and adolescent report, higher scores indicated lower self-control in the adolescent. In wave 3, low self-control was measured using ten items self-reported by the adolescent. Sample items include “You like to take risks,” and “You often try new things just for fun or thrills, even if most people think they are a waste of time.” All items were measured on a five-point Likert scale with higher values indicating lower self-control.

#### Early sexual debut

Early sexual debut was measured in wave three to give sufficient time for the youngest *Add* Health respondents to age beyond the timing for normative sexual debut. Respondents who reported they had ever had sexual intercourse were asked, “How old were you the first time you ever had vaginal intercourse”. Early sexual debut was defined as first vaginal intercourse at prior to 15 years of age [4, 23, 24].

#### Risky sexual behavior

Risky sexual behavior in early adulthood was measured using the three available items from wave four when *Add* Health respondents were 24 to 32 years of age. The items include “Total number of male and female partners in the past 12 months,” “In the past 12 months have you paid someone for sex?” and “In the past 12 months have you had sex with more than one person at about the same time?”

#### Controls

We controlled for sex, age, race, wave one socio-economic stress, wave four socio-economic stress, and wave four relationship status, including married, cohabitating, dating, and single. Wave 1 socio-economic stress was a composite variable consisting of whether or not the participant’s primary responding parent was married, whether or not the family was receiving public assistance, and parent education status. Wave 4 socio-economic stress was comprised of respondent’s educational status, whether or not the participant owned a home, and whether the respondent could meet their basic needs.

##### Analytic strategy

Data were cleaned and prepared in SAS (ver 9.4, SAS Institute Inc., Cary, NC, USA). Frequencies and proportions were calculated to describe the sample. We conducted confirmatory factor analysis (CFA) for each construct of interest with more than two items (waves 1 & 3 self-control and wave 4 risky sexual behavior) in a structural equation modeling (SEM) framework using Mplus Version 7 (Muthén & Muthén, 1998–2012). Items with factor loadings < 0.40 were dropped. We assessed model fit using the following fit indices and cut-offs: Comparative Fit Index (CFI) ≥ 0.95 indicated good fit and < 0.90 indicated poor fit; Root Mean Square Error of Approximation (RMSEA) ≤ 0.06 indicated good fit and > 0.10 indicated poor fit [25].

After defining the measurement model, we used SEM to assess the relationships of interest. SEM is preferable over traditional longitudinal regression analyses because it accounts for measurement error and allows multiple relationships to be measured simultaneously. It is also an ideal approach for testing mediation and moderation. We fit a base model using early sexual debut and self-control (wave 3) as predictors of risky sexual behavior (wave 4), while controlling for the effects of prior self-control (wave 1). After fitting the base model, sociodemographic control variables were added to the model.

We next constructed a mediation model to examine whether early sexual debut and wave 3 self-control mediated the relationship between low self-control at baseline (wave 1) and risky sexual behavior (wave 4). Mediation was assesed by examining the significance of indirect effects using 5000 bootstraps [26]. Following the test for mediation, we created an interaction term between self-control (wave 3) and risky sexual behavior (wave 4) to examine whether self-control moderated the relationship between early sexual debut and later risky sexual behavior.

Finally, we sought to determine if the associations observed in the baseline, mediation and moderation models were different for males and females. We began by assessing measurement invariance for both self-control (wave 3) and risky sexual behavior (wave 4) by testing for uniform differential item functioning (DIF) [27, 28]. DIF indicates that at the same underlying level of self-control or early sex, women scored significantly different than expected compared to men on individual items and the difference was uniform. To examine DIF, we regressed the latent wave 3 self-control and risky sex constructs on gender, and looked for modification indices for individual items (that comprised the wave 3 self-control and risky sex scales) on gender that were ≥ 4.0. If there were modification indices above 4.0, these regression paths were added to the model in stepwise fashion and a chi-square difference test was performed. If the model paths and chi-square difference test were significant, there was evidence of DIF. We adjusted our models for significant differences found for any of the item indicators to ensure that any differences in model relationships observed between males and females were not attributable to DIF.

Because there was evidence that the wave 3 self-control construct was not invariant across genders, we did not test whether the final models significantly varied by gender. Instead, we ran separate final models for males and females to understand the key model relationships for each gender, though we cannot statistically compare these models by gender.

We used the same fit indices and cutoffs for model fit of the structural models as we did for confirmatory factor analyses. In all models, we accounted for the complex survey design (probably weights and clustering). We used the robust weighted least squares maximum likelihood estimator, which is appropriate for data with categorical indicators, to estimate all models. Missing data were addressed using Full Information Maximum Likelihood (FIML).

## Results

### Descriptive data

The sample was comprised of 5734 persons and was 53% female. The average age of all respondents at wave 1 was 16 years old. The majority of respondents were white (58.5%). Approximately 17% of respondents reported they were less than 15 years of age at first sexual intercourse. Sample demographics by sex are reported in Table 1.

**Table 1:** Descriptive Characteristics

	Total (n = 5734)	Male (n = 2702)	Female (n = 3032)
Age in years M (SD)	16.0 (1.75)	16.1 (1.75)	15.9 (1.74)
	n (%)		
Race/Ethnicity			
White Alone	3352 (58.46)	1593 (58.96)	1759 (58.01)
Black Alone or with any other race	1394 (24.31)	631 (23.35)	763 (25.16)
Hispanic	623 (10.87)	296 (10.95)	327 (10.78)
Other, Non-Hispanic	365 (6.37)	182 (6.74)	183 (6.04)
Relationship Status in Wave 4			
Married	2162 (37.70)	885 (32.75)	1277 (42.12)
Cohabitating	1003 (17.49)	475 (17.58)	528 (17.41)
Dating	793 (13.83)	430 (15.91)	363 (11.97)
Single	535 (9.33)	265 (9.81)	270 (8.91)
Wave 1 Socioeconomic Risk			
0 Risks	2997 (60.67)	1422 (60.72)	1575 (60.64)
1 Risk	1429 (28.93)	685 (29.25)	744 (28.65)
2 Risks	403 (8.16)	188 (8.03)	215 (8.27)
3 Risks	110 (2.23)	47 (2.01)	63 (2.43)
Wave 4 Socioeconomic Risk			
0 Risks	1618 (31.94)	704 (30.27)	914 (33.36)
1 Risk	2386 (47.10)	1158 (49.82)	1228 (44.81)
2 Risks	904 (17.84)	386 (16.60)	518 (18.91)
3 Risks	157 (3.10)	77 (3.31)	80 (2.92)
Age at First Intercourse			
Early Age (< 15 years)	954 (16.64)	459 (16.99)	495 (16.33)
Normative Age (≥15 years)	4780 (83.36)	2537 (83.67)	2537 (83.67)

### Measurement model

We created latent variables for risky sexual behavior (wave 4) (factor loadings ranging from 0.61 to 0.91), low self-control (wave 3) (factor loadings ranging from 0.43 to 0.73), baseline low self-Control – self-report (wave 1; factor loadings ranging from 0.56 to 0.79), and baseline low self-control – parent report (wave 1; factor loadings ranging from 0.55 to 0.75). Model fit indices were all above the minimum cutoffs.

### Mediation model

**Fig. 1:**
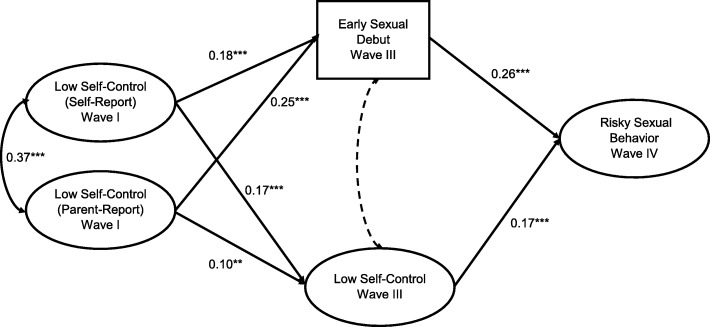
Mediation Model for the relationship between self-control, impulsivity, early sexual debut and risky sexual behavior among male and female respondents to *Add Health*, *n* = 5734. *p < .05, **p < .01, ***p < .001, Model Fit: RMSEA: 0.03; CFI: 0.92; Model Controls for DIF. Dotted line indicates non-significant relationship. *Indirect Paths:* Low Self Control (Self-Report) ➔ Early Sexual Debut ➔ Risky Sexual Behavior: *z* = 4.14, *p* < .001, Low Self Control (Parent Report) ➔ Early Sexual Debut ➔ Risky Sexual Behavior: *z* = 6.07, *p* < .001, Low Self Control (Self-Report) ➔ Impulsivity ➔ Risky Sexual Behavior: *z* = 4.16, *p* < .001, Low Self Control (Parent Report) ➔ Impulsivity ➔ Risky Sexual Behavior: *z* = 3.54, *p* < .001

Figure 1 includes the results of the mediation model for the full sample (Model Fit: RMSEA = 0.03, CFI = 0.92). Early sexual debut (0.26, *p* < .001) and wave 3 low self-control (0.17, *p* < .001) both predicted risky sexual behavior in wave 4. Both adolescent self-report and parent report of baseline low self-control (wave 1) predicted higher likelihood of engaging in early sex and lower self-control at wave 3. Early sexual debut mediated the relationship between wave 1 self-report low self-control and wave 4 risky sex (*z* = 4.14, *p* < .001) and between wave 1 parent report of adolescent self-control and wave 4 risky sex (*z* = 6.07, *p* < .001). Wave 3 self-control mediated the relationship between wave 1 self-report low self-control and wave 4 risky sex (*z* = 4.16, *p* < .001) and between wave 1 parent report of adolescent self-control and wave 4 risky sex (*z* = 3.54, *p* < .001).

### Moderation model

**Fig. 2:**
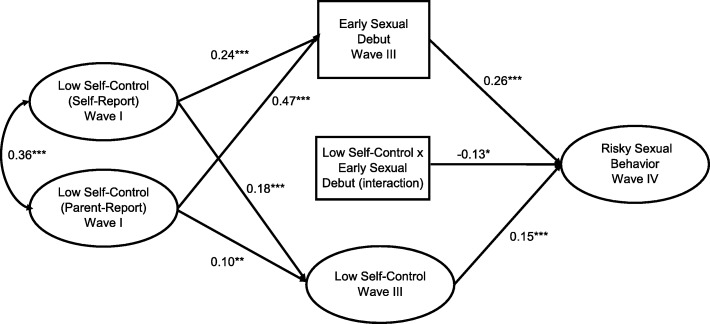
Moderation Model for the effect of impulsivity and early sexual debut on risky sexual behavior among male and female respondents to *Add Health*, n = 5734. *p < .05, **p < .01, ***p < .001. Model Controls for DIF

We next added an interaction term to the model to assess if wave 3 self-control moderated the relationship between early sexual debut and later risky sexual behaviors (Fig. 2). In the full sample, there was evidence of a moderating effect. Unexpectedly, the relationship between early sexual debut and later risky sexual behaviors was stronger among those with higher self-control (wave 3) compared to those who reported lower self-control (− 0.13, *p* < .05).

### Results by gender

For both males and females, early sexual debut and self-control (wave 3) mediated the relationship between baseline low self-control and later risky sexual behaviors. In the female only model, early sex mediated the relationship between parent report of adolescent low self-control and risky sex (*z* = 5.08, *p* < .001) and between adolescent report of low self-control and risky sex (*z* = 2.05, *p* < .05). Wave 3 self-control mediated the relationship between parent report of low self-control (*z* = 2.30, *p* < .05) and adolescent report of low self-control (*z* = 2.31, *p* < .05) with risky sexual behaviors. In the model of only male participants, early sex mediated the relationships between parent report of low self-control (*z* = 2.92, *p* < .01) and adolescent report of low self-control (*z* = 3.04, *p* < .01) with risky sexual behaviors. Wave 3 self-control also mediated the relationships between baseline parent report of low self-control (*z* = 1.99, *p* < .05) and baseline adolescent report of low self-control (*z* = 3.15, *p* < .01).

**Fig. 3:**
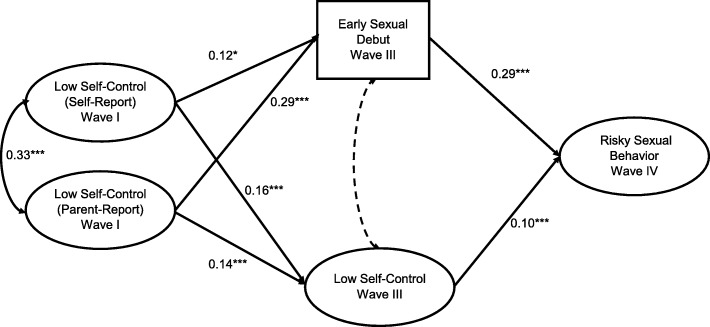
Mediation Model for the relationship between self-control, impulsivity, early sexual debut and risky sexual behavior, among female respondents to *Add Health, n* = 3032. *p < .05, **p < .01, ***p < .001, Model Fit: RMSEA: 0.02; CFI: 0.94, Model Controls for DIF. Dotted line indicates non-significant relationship. *Indirect Paths:* Low Self Control (Self-Report) ➔ Early Sexual Debut ➔ Risky Sexual Behavior: *z* = 2.05, *p* < .05. Low Self Control (Parent Report) ➔ Early Sexual Debut ➔ Risky Sexual Behavior: *z* = 5.08, *p* < .001. Low Self Control (Self-Report) ➔ Impulsivity ➔ Risky Sexual Behavior: *z* = 2.31, *p* < .01. Low Self Control (Parent Report) ➔ Impulsivity ➔ Risky Sexual Behavior: *z* = 2.30, *p* < .01

**Fig. 4:**
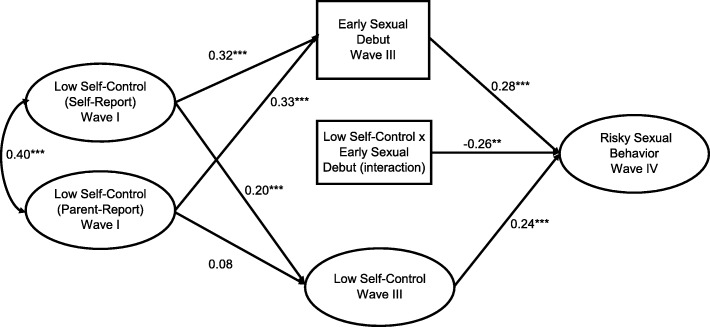
Moderation Model for the effect of impulsivity and early sexual debut on risky sexual behavior among male respondents to *Add Health*, *n* = 2702. *p < .05, **p < .01, ***p < .001. Model Controls for DIF

There was evidence of moderation in the male-only model (− 0.26, *p* < .01), but not in the female-only model. Given these results, the mediation model appears most appropriate for females (Fig. 3) and the model that includes an interaction term is most appropriate for males (Fig. 4).

## Discussion

Approximately 17% of adolescents in our sample reported being less than 15 years of age at first sexual intercourse. This is similar to estimates from other national samples [4, 6]. Consistent with our hypotheses, we observed that low self-control was associated with early sexual debut, supporting the idea that low self-control in early adolescence at least partially explains selection into early sexual debut. Further, we observed that wave 3 low self-control was associated with risky sexual behavior in young adulthood. Additionally, the finding that low self-control was persistent across time with wave 1 self-control being predictive of wave 3 self-control further supports the contribution of adolescent low self-control on risky sexual behavior in adulthood. This suggests that interventions targeting self-control may be influential in delaying entry into sexual intercourse and reducing participation in risky sexual behaviors in adulthood among those with early debut.

Mental contrasting is a learnable skill that helps adolescents visualize their goals; identify potential barriers to reaching these goals, and to develop a plan to overcome these barriers thus increasing self-control. Interventions focusing on capacity building for self-control, such as mental contrasting training, may be effective in helping young adolescents delay entry into sexual activity and/or avoid risky sexual behaviors in adulthood. Duckworth and colleagues found that adolescents who received training on mental contrasting were better able to complete high-stakes tests compared to their peers who did not receive this training. Relating this to early sexual debut and self-control, adolescents who learn mental contrasting may be better able to resist sex in high-pressure social situations if they have first formulated a goal to delay sex and a plan for overcoming likely obstacles [29].

In males, higher self-control strengthened the relationship between early sex and risky sex, which was the opposite of what we expected. This may be due to differences in behavioral expectations surrounding sex between males and females. Sexual script theory [16] identifies gendered prescriptions for socially acceptable sexual behavior. Sexual scripts are generally thought to promote a higher level of sexual activity in males than in females resulting in relatively higher social costs for sexually active females and relatively higher social rewards for sexually active males [18]. Given these sexual scripts, it may be that young adult males with low self-control are more likely to participate in risky sexual behavior regardless of their timing of sexual debut.

These data should be considered with several caveats. This study relies on self-report measures for self-control, early sex and risky sex. Self-report of executive functions such as self-control may be biased. The use of parental reports where available may reduce bias in the study introduced by self-report of self-control. Previous research has acknowledged the challenge of accurately measuring sexual behavior [30, 31]. We acknowledge that the use of biological age at sexual debut is limited and does not consider important issues related to sexual readiness [32]. Further, questions about sexual initiation in *Add* Health are specific to vaginal intercourse and therefore do not appropriately represent participation in other sexual behaviors, including among those without opposite-sex sexual experience. As such, the results of this study cannot be applied to those who identify as non-heterosexual. The gender-specific models in this paper are based on biological sex and likely do not represent persons who are transgender or have a non-binary gender identity. The use of two different measures of self-control in waves 1 and 3 may have introduced measurement error. Further, we acknowledge the possibility that sexual debut for some respondents may have occurred prior to our wave 1 measures of self-control. Our measures of risky sexual behavior in wave 4 was limited to the three questions available in the data and as such, does not capture all forms of risky sexual behavior.

Despite these limitations, this study has a number of strengths. The use of a longitudinal sample stands in contrast to most studies of early sex and risky sex, which have relied on cross-sectional data. The data were analyzed using structural equation modeling which accounts for measurement error and provides an ideal forum for examining mediation and moderation. Finally, the national representative *Add* Health sample allows wide generalizability of the findings of this study.

## Conclusion

This study adds to the body of literature suggesting the role of executive functions such as self-control in sexual behaviors including early sexual debut and risky sexual behavior in adulthood. Previous research suggests that interventions may be useful in increasing executive function, which may have an impact in delaying sexual debut or ensuring that the timing of sexual debut is developmentally appropriate for individuals based on social and emotional readiness and reducing participation in risky sexual behaviors in young adulthood.

## Data Availability

The data used in this study is publicly available at: https://www.cpc.unc.edu/projects/addhealth/

## Abbreviations

CFA: Confirmatory factor analysis
CFI: Comparative fit index
DIF: Differential item functioning
FIML: Full information maximum likelihood
RMSEA: Root mean square error of approximation
SCT: Social cognitive theory
SEM: Structural equation modelling
STD: Sexually transmitted disease
